# Haemodialysis is an effective treatment in acute metabolic decompensation of maple syrup urine disease

**DOI:** 10.1016/j.ymgmr.2015.07.001

**Published:** 2015-07-10

**Authors:** P.S. Atwal, C. Macmurdo, P.C. Grimm

**Affiliations:** a Stanford University, Dept. of Pediatrics, Div. of Med. Genetics, 300 Pasteur Drive, Stanford, CA, 94305, United States; b Stanford University, Dept. of Pediatrics, Div. of Nephrology, 300 Pasteur Drive, Stanford, CA, 94305, United States

**Keywords:** BCAA, branched chain amino acids, BCKD, branched-chain-ketoacid dehydrogenase, MSUD, maple syrup urine disease, Maple syrup urine disease, Dialysis, Haemodialysis, Acute metabolic crisis

## Abstract

Acute metabolic decompensation in maple syrup urine disease can occur during intercurrent illness and is a medical emergency. A handful of reports in the medical literature describe the use of peritoneal dialysis and haemodialysis as therapeutic inventions. We report the only patient from our centre to have haemodialysis performed in this setting. Combined with dietary BCAA restriction and calorific support, haemodialysis allows rapid reduction in plasma leucine concentrations considerably faster than conservative methods.

## 1 Introduction

Maple syrup urine disease (MSUD) is caused by decreased activity of branched-chain-ketoacid dehydrogenase (BCKD), the second enzyme in the pathway for the degradation of the three branched-chain amino acids (BCAAs), leucine, isoleucine and valine [1]. The resultant metabolic block leads to elevated plasma concentrations of BCAAs and leads to encephalopathy with lethargy, opisthotonus, stereotyped movements such as “bicycling”, respiratory failure and death if left untreated due to acute leucine intoxication. A maple syrup odour can be detected in cerumen shortly after birth and urine around days 5–7 of life in affected patients [1], [2], [3]. In addition, there is evidence that alpha-ketoisocaproic acid is a major neurotoxin contributing to the encephalopathic syndrome.

Acute metabolic decompensation can occur during intercurrent illness and is a medical emergency. Prompt reduction of plasma levels of BCAAs is required to prevent serious complications, including decreased intellectual performance and also brain edema with resultant central transtentorial herniation which is almost always lethal [4]. Conservative approaches include withholding BCAAs in the diet, supplementation of valine and isoleucine and providing surplus calories to promote anabolism [1]. Efficient renal tubular reabsorption of filtered amino acids results in low rates of renal clearance of BCAAs thus inhibiting rapid reduction in plasma levels using conservative approaches, although it is noted that the mainstay of conservative approaches is sustained positive rate or net protein accretion [5]. A handful of reports in the medical literature describe the use of peritoneal dialysis and haemodialysis as therapeutic inventions for rapid reductions in plasma BCAA levels, with limited data on presentation and outcomes [6]. Haemodialysis in particular allows for rapid and high clearance to be delivered [2].

We report the only patient from our centre to have haemodialysis performed as a treatment in this setting and their outcome. Our patient was a 4 year old ex-full term female born to a G4P2 mother with known mild MSUD diagnosed by newborn screening. Family history was mixed Mexican and African-American and consanguinity was denied. She was obtunded on presentation with a 5 day history of fever, vomiting and reduced oral intake. Her admission leucine level was 1959 nmol/ml (normal range 49–216) and her neurological exam demonstrated no purposeful movements and withdrawal to pain only. Glasgow coma scale was 7/15 and CT brain showed signs of cerebral edema. Initial serum testing included sodium of 136 meq/l, bicarbonate of 14 meq/l, and serum potassium of 3.4 meq/l, with an anion gap of 17 meq/l.

## 2 Case report

We report the only patient from Stanford University Medical Center to have haemodialysis performed as a treatment in this setting and their outcome. Our patient was a four-year-old, ex-full term female born to a G4P2 mother with known mild MSUD diagnosed by newborn screening. Our patient's ethnicity was Mexican/African-American, and parents denied consanguinity. She was obtunded on presentation with a five-day history of fever, vomiting and reduced oral intake. Her admission leucine level was 1959 nmol/ml (normal range 49–216), valine 1225 nmol/ml (normal range 74–312), and isoleucine of 900 nmol/ml (normal range 22–107). Initial serum testing included sodium of 136 meq/l, bicarbonate of 14 meq/l, and serum potassium of 3.4 meq/l, with an anion gap of 17 meq/l. Her neurological exam demonstrated no spontaneous purposeful movements, and she withdrew to pain only. Her Glasgow coma scale was 7/15, and a CT of the brain showed signs of cerebral edema.

After a careful consideration of risks and benefits, haemodialysis was chosen to quickly reduce the leucine level. A femoral vein catheter was inserted using the Seldinger Technique for vascular access. Sedation was not required due to the patient's reduced neurological status. Haemodialysis was performed using an F5 Dialyzer (Fresenius Medical Care), with an initial blood flow of 100 ml/min and dialysate flow of 500 ml/min, a high sodium dialysate of 145 meq/l was chosen to reduce osmolality shifts and reduce risk of exacerbating brain edema. Dialysate potassium was 4 meq/l and bicarbonate was 40 meq/l. The initial run terminated after 90 min due to circuit clotting. Haemodialysis was reinitiated with a new circuit and increased blood flow of 130 ml/min for another 90 min for a total of 3 h of haemodialysis. Mannitol 0.25 mg/kg was given at the initiation of dialysis to mitigate osmolal shifts.

Calculations:

Qb = blood flow

Hct = haematocrit

Leu_1_ = pre dialyzer leucine level

Leu_2_ = post dialyzer leucine level.

Clearance of leucine

(1) $$\left(1-\left(\frac{\mathrm{Leu}2}{\mathrm{Leu}1}\right)\right)\times 100\%=\mathrm{Leucine}\;\mathrm{Clearance}.$$

Plasma clearance of leucine can be calculated by the following formulae

(2) $$\mathrm{Qb}\times \left(100-\mathrm{Hct}\right)=\mathrm{dialyzer}\;\mathrm{plasma}\;\mathrm{flow}$$

(3) Dialyzerplasmaflow×LeucineClearance=LeucinePlasmaClearance

(4) $$\mathrm{Dialyzer}\;\mathrm{plasma}\;\mathrm{flow}\times \left(\mathrm{Leu}1-\mathrm{Leu}2\right)=\mathrm{Total}\;\mathrm{Body}\;\mathrm{Leucine}\;\mathrm{Clearance}\;\mathrm{in}\;\mathrm{nmol}\;\mathrm{per}\;\mathrm{minute}.$$

### 2.1 Summary of results

At initiation of HD the leucine level was 1685 nmol/ml. A post dialysis filter leucine level was 374 nmol/ml. The pre-dialyzer haematocrit was 30.5%.

Leucine clearance was (Eq. (1))

$$\left(1-\left(\frac{374}{1685}\right)\right)\times 100\%=77.8\%.$$

With an achieved blood flow of 100 ml/min, the dialysis Rx achieved 77.8% clearance of the blood processed.

Dialyzer plasma flow (Eq. (2))

$$100\times \left(100-30.5\right)=69.5\;\mathrm{ml}\;\mathrm{per}\;\text{minute}.$$

Leucine Plasma Clearance (Eq. (3))

69.5×77.8%=54mlperminute.

Total Body Leucine Clearance (Eq. (4))

$$69.5\times \left(1685-374\right)=91,114\;\text{nmol}\;\mathrm{per}\;\text{minute}.$$

These calculations indicate that at the initiation of haemodialysis, 91.1 μmol of leucine was removed per minute of dialysis. As the plasma leucine level fell, the rate of removal would fall in parallel.

**Fig. 1:**
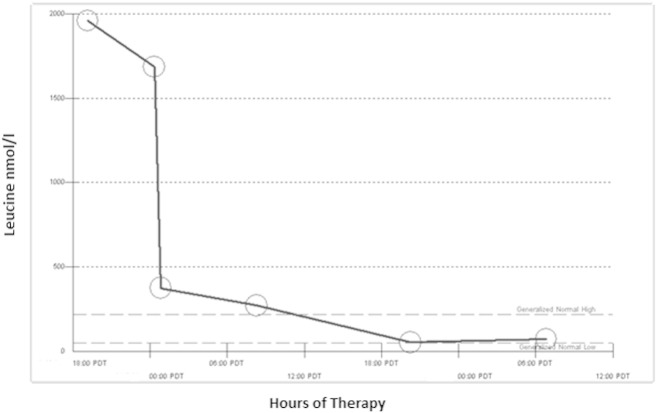
Acute leucine level trend post-initiation of haemodialysis.

Within 6 h of initiation of haemodialysis a peripheral blood leucine level of 374 nmol/ml was achieved (see Fig. 1). Neurological status had also improved significantly and within 12 h the patient was opening eyes spontaneously and able to obey commands. At 24 h neurological status further improved and she was back to neurological baseline on day two.

## 3 Discussion

We report the only case of haemodialysis used in the treatment of acute MSUD crisis from our centre; currently there are only a handful of case reports in the medical literature, with very limited data on success rates and outcomes. This case report is notable because of 3 things. The first is that the patient is 4 years old. Most other patients reported in the literature requiring dialysis are newborns or infants [4]. The second contribution to the literature of our report is the detailed clearance calculations obtained from the procedure, showing the high efficiency of haemodialysis for rapidly clearing leucine and allowing future comparisons by other investigators using this or other modalities. Finally, we demonstrate success of the approach of administering mannitol and dialyzing against hypertonic saline to prevent worsening of cerebral edema. There is recognition that haemodialysis may aggravate cerebral edema by inducing osmolal shifts [5], [6]. Our patient had CT evidence of cerebral edema along with a severe reduction in level of consciousness prior to initiation of haemodialysis. The fact that she recovered so quickly shows the safety of this approach.

Our results show that, combined with dietary BCAA restriction and calorific support, haemodialysis is a highly effective treatment that allows rapid reduction in plasma leucine concentrations considerably faster than conservative methods. Given the relatively low molecular mass of BCAA, it has been reported that their clearance rate is roughly similar to urea [2]. We postulate that this rapid reduction in leucine levels reduced the risk of further complications such as long-term neurological sequelae and shortened our patient's hospital stay. In addition we note isolated reports of ‘rebound’ hyperleucinema 2–3 days post-haemodialysis however did not observe this in our patient. Caution, however, should be exercised with haemodialysis due to the theoretical risk of exacerbating existent brain edema by fluid shift with the rapid reduction in the leucine level. Due to these risks, we recommend that haemodialysis only be performed in a centre of excellence with ready availability of specialists in biochemical genetics and nephrology. We note that this was the first time our patient presented in acute metabolic crisis and had been deemed a ‘mild’ case of MSUD therefore this case also serves as a reminder that even these cases can have severe decompensation if left untreated. In addition we hope that our methods may prove as a useful guide for physicians considering this haemodialysis in the context of acute decomposition of MSUD. In summary, haemodialysis should be considered in MSUD patients presenting with severely altered mental status and concern for cerebral edema.

## Disclosure

None.

## Compliance with ethics guidelines

All authors declare that they have no conflict of interest.

## Informed consent

All procedures followed were in accordance with the ethical standards of the responsible committee on human experimentation (institutional and national) and with the Helsinki Declaration of 1975, as revised in 2000 (5). Informed consent was obtained from all patients for being included in the study.
